# Acquisition of Cry1Ac Protein by Non-Target Arthropods in *Bt* Soybean Fields

**DOI:** 10.1371/journal.pone.0103973

**Published:** 2014-08-11

**Authors:** Huilin Yu, Jörg Romeis, Yunhe Li, Xiangju Li, Kongming Wu

**Affiliations:** 1 State Key Laboratory for Biology of Plant Diseases and Insect Pests, Institute of Plant Protection, Chinese Academy of Agricultural Sciences, Beijing, China; 2 Institute for Sustainability Sciences ISS, Agroscope, Zurich, Switzerland; Institute of Vegetables and Flowers, Chinese Academy of Agricultural Science, China

## Abstract

Soybean tissue and arthropods were collected in *Bt* soybean fields in China at different times during the growing season to investigate the exposure of arthropods to the plant-produced Cry1Ac toxin and the transmission of the toxin within the food web. Samples from 52 arthropod species/taxa belonging to 42 families in 10 orders were analysed for their Cry1Ac content using enzyme-linked immunosorbent assay (ELISA). Among the 22 species/taxa for which three samples were analysed, toxin concentration was highest in the grasshopper *Atractomorpha sinensis* and represented about 50% of the concentration in soybean leaves. Other species/taxa did not contain detectable toxin or contained a concentration that was between 1 and 10% of that detected in leaves. These Cry1Ac-positive arthropods included a number of mesophyll-feeding Hemiptera, a cicadellid, a curculionid beetle and, among the predators, a thomisid spider and an unidentified predatory bug belonging to the Anthocoridae. Within an arthropod species/taxon, the Cry1Ac content sometimes varied between life stages (nymphs/larvae *vs.* adults) and sampling dates (before, during, and after flowering). Our study is the first to provide information on Cry1Ac-expression levels in soybean plants and Cry1Ac concentrations in non-target arthropods in Chinese soybean fields. The data will be useful for assessing the risk of non-target arthropod exposure to Cry1Ac in soybean.

## Introduction

Genetically modified (GM) crops expressing *cry* genes from *Bacillus thuringiensis* (*Bt*) are widely used to control major insect pests and are an important component of integrated pest management (IPM) systems [1]–[4]. The damage caused by lepidopteran pests greatly reduces soybean yield and quality [5]. Recently, Monsanto Company has developed an insect-resistant transgenic soybean cultivar called MON87701. This soybean line expresses the *cry1Ac* gene and exhibits excellent efficacy against some lepidopteran pests [6], [7].

Before a new GM plant is grown in the field, its potential for harming valuable non-target organisms (NTO) is determined as part of an environmental risk assessment [8]–[11]. Risk is a function of hazard (here: toxicity of the insecticidal compound) and the likelihood that this hazard will be realized (here: likelihood of exposure to hazardous concentrations of the insecticidal compound) [8], [9]. Knowledge about the NTOs most likely to be exposed to the insecticidal compound enables researchers to determine which species should be the focus of risk assessment [12]–[14].

Herbivores are directly exposed to *Bt* proteins when feeding on transgenic plant tissues [15]. The quantity of *Bt* protein ingested can differ widely among herbivore species. This variation reflects the time and site of toxin expression in the plant, the feeding ecology of the herbivores, and the amount of plant material that is ingested [15]–[17]. For example, the two-spotted spider mite, *Tetranychus urticae* (Acari: Tetranychidae), has been reported to contain high concentrations of Cry proteins when fed *Bt* maize or *Bt* cotton; the concentrations were equal to or even higher than the levels in the plant tissues [18]–[20]. In contrast, larvae of Lepidoptera like *Helicoverpa amigera* (Noctuidae) or *Spodoptera littoralis* (Noctuidae) contain Cry protein levels that are one or two orders of magnitude lower than those in *Bt* plant tissues [21], [22]. Interestingly, sucking pests such as aphids and planthoppers were reported to contain no or only trace amounts of Cry proteins after feeding on *Bt* crops [23], [24].

Predators are exposed to *Bt* toxins mainly by consuming herbivores that have ingested the toxin [15]. Omnivorous species can also acquire Cry proteins by directly feeding on pollen or other *Bt* plant tissue. This has, for example, been reported for various species of Heteroptera, such as *Orius* spp. (Anthocoridae), adult *Chrysoperla* spp. (Neuroptera: Chrysopidae), and species of ladybird beetles (Coleoptera: Coccinellidae) [25]–[29]. Tri-trophic laboratory studies have revealed that various species of predators belonging to different arthropod orders contain Cry proteins after feeding on prey reared on *Bt*-transgenic maize, rice, or cotton plants [15], [19], [27], [30]–[40]. In general, the concentrations of *Bt* proteins were significantly diluted when transferred to higher trophic levels, and there has been no indication of toxin accumulation, which is also consistent with field studies with different *Bt* plants [30], [40], [41]–[45].

The objective of our study was to characterize the level at which different arthropod species are exposed to the Cry protein when foraging in *Bt* soybean fields. The larger goal was to identify non-target species that are most likely at risk as a consequence of such exposure. These data will provide baseline information for further non-target risk assessment of transgenic *Bt* soybean.

## Materials and Methods

### Ethics statement

No specific permits were required for the described field studies. The soybean fields from which the arthropods used in this study were originally collected were owned by the authors' institute (Institute of Plant Protection, Chinese Academy of Agricultural Sciences, CAAS). These field studies did not involve endangered or protected species.

### Plants

Experiments were conducted with transgenic soybean MON87701, which produces the Cry1Ac protein (*Bt*), and the corresponding non-transformed near-isoline A5547. Soybean seeds were supplied by Monsanto Company (St. Louis, MO, USA). In 2010, the two soybean lines were sown at the Agriculture Experiment Station of the Chinese Academy of Agriculture Sciences (CAAS) located at Langfang, Hebei Province. Soybeans were managed according to the common growing practices in the region but without pesticide application. *Bt* soybean and control soybean were grown in separate fields at a distance of about 500 m. Each field was divided into four 180-m^2^ plots (length×width: 15 m×12 m). Plots were isolated by belts of maize plants (length×width: 5 m×12 m).

### *Bt* protein content in transgenic soybean plants

Different soybean plant tissues were collected at different growth stages in 2010: before anthesis (V6–8 and V11–12; 1 to 25 August); during anthesis (R1 and R3; 26 August to 10 September); and after anthesis (R5, R6, and R7; 15 September to 10 November) [46], [47]. Only leaves were collected before anthesis; leaves and flowers were collected during anthesis; and leaves and pods were collected after anthesis. At each sampling date, 30 leaves or 50 flowers or 20 pods were collected from 10 randomly selected soybean plants from each of the four soybean plots as one sample, resulting in a total 44 samples [leaves: 4 replications (plots)×7 growth stages, resulting in 28 samples; flowers: 4 replications (plots)×1 growth stage (anthesis), resulting in 4 samples; pods: 4 replications (plots)×3 growth stages (R5, R6, R7), resulting in 12 samples] for each *Bt* soybean and control soybean. The leaves were the third fully expanded trifoliate leaves, and the flowers were taken from the upper part of the plants. All samples were kept at −80°C for later ELISA analyses.

### Cry protein content in arthropod species collected from *Bt* soybean plots

Arthropods were collected from soybean plots using a sweep net between 5 August and 30 October 2010 when the soybean plants were at the V6 to R7 growth stage. In addition, leaves covered with aphids were cut, put into a plastic bag, and transported to the laboratory; soybean aphids were collected from the underside of the leaves using a camel-hair brush and amicroscope. Immediately after they were collected in the field, all other arthropods were placed individually in 2- or 5-ml centrifuge tubes and were kept in a cooling box to reduce metabolism and to reduce excretion of *Bt* protein. Once transferred to the laboratory, the arthropods were immediately stored in a −80°C freezer. Since the experimental plots were not large enough, and the arthropods were not evenly distributed, for some species, we could not collect enough individuals for ELISA measure in some plots, while in other plots excess individuals were collected. In addition, ELISA measures showed that *Bt* concentrations in samples collected from different field plots were not significantly different. Therefore, we pooled arthropod individuals of the same species collected from the different plots and subsequently divided them in equal sub-samples for the ELISA analyses. The arthropod species that were analysed are listed in Tables 1 and 2 and in Table S1. Most species names were verified using the Catalogue of Life (www.catalogueoflife.org) and Fauna Europaea (www.faunaeur.org). Species names that were not included in the databases were confirmed by experts from China Agriculture University and Northwest A&F University.

**Table 1:** Cry1Ac concentrations in arthropods collected in *Bt* soybean before, during, and after anthesis in 2010.

Order	Family	Species	Functional group^a^	Stage	Mean dry weight per individual [mg]	Mean Cry1Ac concentration [µg/g DW] (number of individuals per sub-sample)^b^		
						Before anthesis	During anthesis	After anthesis
**Araneae**	Thomisidae	*Misumenopos tricuspidatus* Fabricius	P	Adult	3.58	0.230 (15)	0.051 (15)	0.210 (15)
				Juvenile	1.24	0.079 (15)	0.121 (15)	0.149 (15)
		Not identified	P	Mix	3.06	n.c.	n.c.	4.247 (8)
**Coleoptera**	Chrysomelidae	*Paraluperodes suturalis nigrobilineatus* Motschulsky	H	Adult	1.47	0.097 (2)	0.091 (2)	0.064 (2)
	Coccinellidae	*Propylea japonica* Thunberg	P	Adult	3.07	<0.004 (15)	<0.001 (15)	<0.007 (15)
				Larva	1.86	0.034 (15)	0.023 (15)	0.034 (15)
	Scarabaeidae	*Anomala cuprea* (Hope)	H	Adult	12.1	<0.003 (3–4)	n.c.	n.c.
**Diptera**	Cecidomyiidae	*Aphidoletes abietis* (Kieffer)	P	Adult	0.40	<0.009 (5)	<0.002 (5)	<0.007 (5)
	Neriidae	Not identified	S	Adult	0.42	n.c.	0.060 (8–11)	0.066 (6–10)
**Hemiptera**	Anthocoridae	*Orius* spp.	P	Mix	0.14	n.c.	0.047 (15)	0.133 (15)
		Not identified	P	Adult	0.26	n.c.	0.282 (15)	0.624 (15)
	Aphididae	*Aphis glycines* Matsumura	H	Mix	0.07	<0.001 (25)	<0.002 (25)	<0.007 (25)
	Cicadellidae	*Cicadella viridis* Linnaeus	H	Adult	6.90	<0.009 (2)	<0.001 (2)	<0.002 (2)
				Nymph	0.28	1.310 (15)	0.054 (15)	0.616 (15)
	Lygaeidae	*Geocoris pallidipennis* (Costa)	P	Mix	0.74	0.304 (7)	0.086 (7)	0.148 (7)
	Miridae	*Deraeocoris punctulatus* (Fallén)	P	Adult	1.01	0.805 (15)	<0.006 (19)	<0.005 (15)
		*Lygus* spp.	H	Adult	2.05	0.115 (15)	0.025 (15)	<0.007 (15)
				Nymph	0.77	2.656 (15)	0.124 (15)	0.066 (15)
		*Trigonotylus ruficornis* (Geoffroy in Fourcroy)	H	Adult	0.66	<0.003 (15)	<0.011 (15)	<0.007 (15)
				Nymph	0.52	<0.005 (15)	<0.007 (15)	<0.011 (15)
	Nabidae	*Nabis stenoferus* Hsiao	P	Adult	2.58	<0.004 (5)	<0.009 (5)	n.c.
				Nymph	0.87	n.c.	0.042 (5)	0.041 (5)
	Pentatomidae	*Dolycoris baccarum* (Linnaeus)	H	Adult	21.8	n.c.	n.c.	0.033 (2)
				Nymph	9.27	0.275 (2–3)	0.042(2)	1.116 (2)
		*Halyomorpha halys* (Stål)	H	Nymph	1.97	n.c.	0.406 (2)	n.c.
	Rhopalidae	*Rhopalus maculates* (Fieber)	H	Adult	8.76	0.031 (5)	<0.002 (5)	0.084 (5)
**Neuroptera**	Chrysopidae	*Chrysoperla* spp.	H	Adult	3.86	0.031 (15)	<0.014 (15)	0.025 (15)
			P	Larva	2.30	0.218 (15)	0.086 (15)	0.265 (15)
**Orthoptera**	Acrididae	*Atractomorpha sinensis* I. Bolivar	H	Adult	86.3	16.240 (1)	8.082 (1)	5.269 (1)
				Nymph	22.0	<0.002 (1)	0.055 (1)	0.070 (1)
	Gryllidae	*Velarifictorus micado* (Saussure)	H	Adult	106	<0.001 (1)	n.c.	n.c.

ELISA results below the limit of detection (LOD) are indicated as ‘<’with the corresponding LOD value. This table only includes values based on the analysis of three sub-samples.

a H– herbivore, P – predator, S – saprophage.

b n.c. – not collected.

**Table 2:** Detection of Cry1Ac in arthropods collected from *Bt* soybean plots at different growth stages for which only one or two sub-samples were analysed (by ELISA).

Cry1Ac	Order	Family: Species [stage analysed^a^]
Detected	Araneae	Linyphiidae: *Erigonidium graminicolum* (Sundevall) [A]
	Coleoptera	Curculionidae: *Xylinophorus mongolicus* Zumpt, T. [ A], *Sympiezomias velatus* Kôno, H. [A]; Chrysomelidae: *Callosobruchus chinensis* (Linnaeus) [A]
	Diptera	Agromyzidae: *Melanagromyza sojae* Zehntner [A]; Drosophilidae: n.i. [A]
	Hemiptera	Alydidae: *Riptortus pedestris* (Fabricius) [N,A]
	Hymenoptera	Apidae: *Apis mellifera ligustica* Spinola [A]
	Lepidoptera	Arctiidae: *Spilosoma niveus* (Ménétriés) [L]; Sphingidae: *Clanis bilineata tsingtauica* Mell [L]
	Odonata	Zygoptera: n.i. [A]
	Orthoptera	Acrididae: *Diabolocatantops pinguis* (Stål) [N,A]
Not detected	Araneae	Lycosidae: *PardosaT-insignita* Bosenberg & Strand [M]
	Coleoptera	Elateridae: *Pleonomus canaliculatus* Falderman [A]; Tenebrionidae: n.i. [A]
	Dermaptera	Anisolabididae: *Euborellia pallipes* Shiraki [A]
	Diptera	Anthomyiidae: *Delia platura* (Meigen) [A]; Calliphoridae: n.i. [A]; Dolichopodidae: n.i. [A]; Syrphidae: *Episyrphus balteatus* (De Geer) [A], *Sphaerophoria* sp. [A]
	Hemiptera	Pentatomidae: *Eysacoris guttiger* (Thunberg) [A]
	Hymenoptera	Braconidae: *Microplitis mediator* (Haliday) [A]; Formicidae: n.i. [A]; Ichneumonidae: *Campoletis chlorideae* Uchida[A]; Sphecidae: n.i. [ A]; Vespidae: n.i. [ A]
	Lepidoptera	Arctiidae: *Spilosoma niveus* (Ménétriés) [A]; Lycaenidae: *Plebejus argus* (Linnaeus) [A]; Pieridae: *Colias poliographus* Motschulsky [A]; Pyralidae: *Dichocrosis punctiferalis* Guenée [A]

a A: adults, L: larvae, M: mixture of all available stages, N: nymphs; n.i. = species not identified.

### ELISA measurement

The concentrations of Cry1Ac in fresh soybean leaves and insect samples were measured by double-sandwich ELISA using the Cry1Ac detection kit from Envirologix Inc. (Portland, ME, USA). Before measurement, the collected arthropods were identified to species and then washed in deionized water to remove any *Bt* protein from their outer surface before lyophilization. For small arthropods, several or many individuals were pooled as a sample. For larger arthropods (e.g., the grasshopper *Atractomorpha sinensis*; Orthoptera: Acrididae), one individual was analyzed per sample. Thus, the dry weight of the arthropod samples ranged from 1.6 to 100 mg. Table 1 and Table S1 provide information about the number of individuals that were pooled per sample. Whenever possible, arthropods were split into three samples that were analysed separately. Arthropod samples were homogenized in phosphate-buffered saline with 0.05%Tween-20 (PBST). The ratio of lyophilized sample (dry weight, DW) to extraction buffer was 20 mg∶1 ml. After PBST was added to the samples in a centrifuge tube, the samples were fully ground with a Tissuelyser II mill from QIAGEN (Germany) (frequency: 28/s, 4 min). After centrifugation at 12000× *g* and appropriate dilution of the supernatants, ELISA was performed according to the manufacturer's instructions. The measured OD values were calibrated to a range of concentrations of purified Cry1Ac protein purchased from Envirotest-China (agent for Envirologix Inc., Portland, ME, USA; www.envirotest-china.com). The protoxin from *B. thuringiensis* had been expressed as single-gene products in *Escherichia coli* at Case Western Reserve University (USA). The protoxin inclusion bodies were then dissolved and trypsinized, and then isolated and purified by ion exchange HPLC; the pure fractions were then desalted and lyophilized. The purity was about 94–96% (Marianne P. Carey, Case Western Reserve University, personal communication). The toxin was considered not detectable if the concentration was lower than three-fold concentrations of blank optical density (about 0.02 µg/g DW).

Samples from various species belonging to the different arthropod orders addressed in the present study that were collected in control soybean plots were also analysed by ELISA to test for any cross-reaction of arthropod proteins with the ELISA. No such cross-reaction was apparent.

### Statistical analyses

For comparison of Cry protein concentrations in soybean tissue at different growth stages, one-way ANOVAs were carried out followed by Tukey's HSD test using SPSS 13.0.

## Results

### *Bt* protein content in transgenic soybean plants

**Figure 1:**
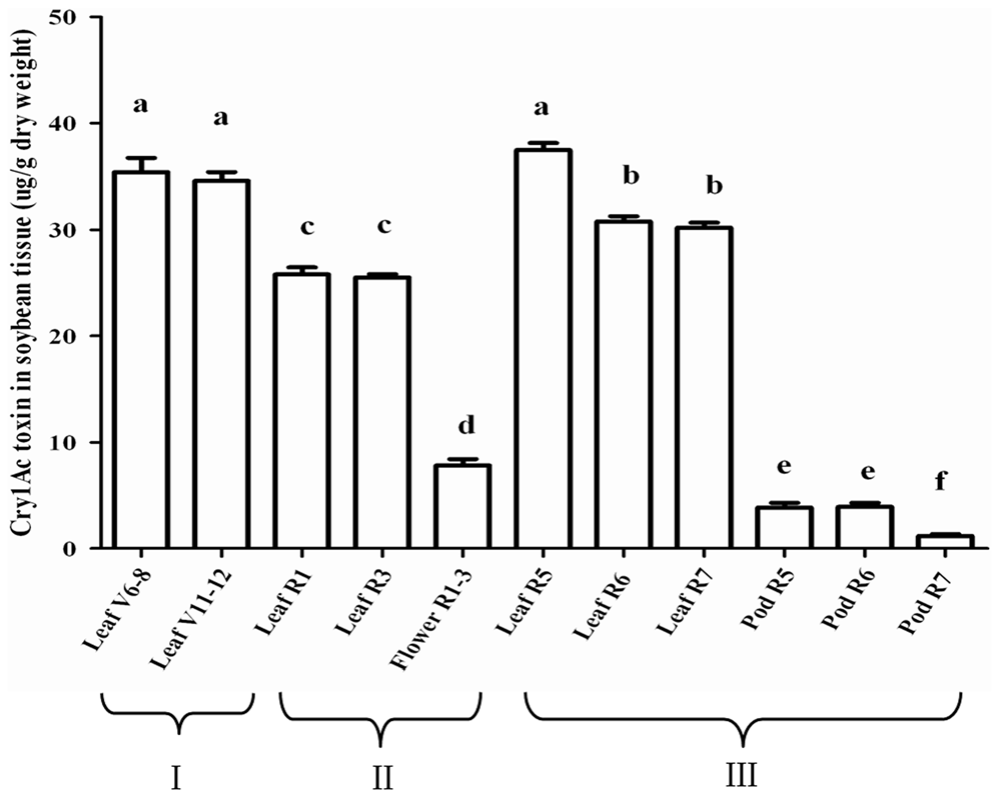
Cry1Ac toxin concentrations (µg/g dry weight, mean+SE) in plant tissues of *Bt* soybean from the field. Samples were taken before (I), during (II) and after anthesis (III) (n = 6). Bars with different letters are significantly different at *P*<0.05.

Concentrations of Cry1Ac differed significantly among different soybean plant tissues collected at different growth stages (one-way ANOVA, F_10,65_ = 314.80, P<0.0001) (Fig. 1). The Cry1Ac content in leaves ranged from 25.50 to 37.50 µg/g DW. Before anthesis, the leaves collected from *Bt* soybean plants at V6–8 and V11–12 had similar Cry1Ac contents (P = 0.70). During anthesis, Cry1Ac levels in leaves significantly declined, i.e., levels were significantly lower at R1 and R3 than at V6–8 and V11–12. After anthesis, the Cry1Ac content rebounded, reaching a level at R5 (37.50 µg/g DW) that was similar to the levels before anthesis. Subsequently, the concentration in leaves declined significantly, but the levels at R6 and R7 remained higher than the levels measured during anthesis. The Cry1Ac concentration was significantly lower in flowers than in any of the leaf samples but was significantly higher than in pod samples (P<0.0001). Cry1Ac contents were similar in pods at R5 and R6 but declined significantly at R7 (P<0.0001). No Cry1Ac was found in control soybean tissues.

### Cry1Ac toxin content in arthropods represented by three samples

In total, we collected and analysed different life stages of more than 50 arthropod species/taxa belonging to 42 families in 10 orders (Tables 1, 2, Table S1). ELISA results for species for which three sub-samples were analysed are presented in Table 1.

Among the species/taxa for which three samples were analysed, a total of 17 were positive for Cry1Ac, i.e., the protein levels were higher than the detection limit (Table 1). For all positive samples, the amounts of Cry1Ac were lower than the amounts measured in soybean leaf tissue (based on a mean level of 32 µg/g DW). By far the highest concentration was detected in adults of the herbivorous grasshopper *A. sinensis*, which contained up to 16.24 µg Cry1Ac/g DW, representing about half of the concentration detected in soybean leaves. For other samples, the species contained between 1 and 10% of the amount of Cry1Ac in soybean leaves, and these species included adult *Misumenopos tricuspidatus* (Araneae: Thomisidae), adults of unidentified Anthocoridae (Hemiptera), adults of *Deraeocoris punctulatus* and nymphs of *Lygus* spp. (both Hemiptera: Miridae), nymphs of *Dolycoris baccarum* and *Halyomorpha halys* (both Hemiptera: Pentatomidae), and nymphs of *Cicadella viridis* (Homoptera: Cicadellidae).

For some species for which different life stages were collected, the quantity of Cry1Ac significantly differed between adults and larvae/nymphs. This was the case for three species of herbivores, including *Lygus* spp. and *C. viridis*, in which Cry1Ac concentrations were generally higher in the nymphs than in adults, and *A. sinensis*, in which levels were higher in adults than in nymphs (Table 1). In the case of *Trigonotylus ruficornis* (Hemiptera: Miridae), Cry1Ac levels in adults and nymphs were always below the detection limit. For predators, two species contained higher concentrations in the larvae than in adults, and these were *Propylea japonica* (Coleoptera: Coccinellidae) and *Chrysoperla* spp. In the case of *Misumenopos tricuspidatus* (Araneae: Thomisidae), levels did not differ between adults and juveniles (Table 1).

For some species, Cry1Ac concentrations differed among the three sampling periods, i.e., before, during, and after anthesis (Table 1). In the case of adults and nymphs of the herbivore *Lygus* spp. and adults of the predator *D. punctulatus*, Cry1Ac concentrations were highest before anthesis. In contrast, Cry1Ac concentrations in nymphs of *D. baccarum* were highest after anthesis. For adults of *Paraluperodes suturalis nigrobilineatus* (Coleoptera: Chrysomelide), Cry1Ac levels were similar before, during, and after anthesis.

Cry1Ac was not detected in any arthropod sample from control soybean plots.

### Cry1Ac toxin content in arthropods represented by one or two samples

Results for species for which only one or two sub-samples were analysed are summarized in Table 2. Rather than presenting mean values for Cry1Ac concentration, Table 2 divides the species into those which were positive or negative for Cry1Ac. Additional details are provided in Table S1. Among species for which analyses were replicated fewer than three times, 12 species/taxa were positive for Cry1Ac (Table 2, Table S1). A significant amount of Cry1Ac (about 13% of that detected in soybean leaves) was detected in unidentified spiders belonging to the Thomisidae. In addition, relatively high amounts of Cry1Ac (1 to 10% of that in soybean leaves) were detected in adults of *Sympiezomias velatus* (Coleoptera: Curculionidae), adults and nymphs of *Riptortus pedestris* (Hemiptera: Alydidae), larvae of *Spilosoma niveus* (Lepidoptera: Arctiidae), and adults of an unidentified species of Zygoptera (Odonata). Nineteen species/taxa contained Cry1Ac levels below the detection limit (Table 2, Table S1).

## Discussion

Throughout the growing season, Cry1Ac protein concentrations in *Bt* soybeans were higher in leaves than in other tissues. The concentrations reached a maximum of 37.50 µg/g DW, which is approximately equivalent to 13.4 µg/g fresh weight (FW) [7]. Thus, the Cry1Ac concentrations in *Bt* soybean leaves were higher than the Cry1A concentrations reported from leaves of field-grown *Bt* cotton (0.7 µg/g FW), *Bt* rice (8 µg/g FW), and *Bt* maize (4 µg/g FW) [48]–[50]. The Cry1Ac concentration in soybean leaves declined significantly during anthesis and then gradually rebounded. A similar expression pattern has been reported for *Bt* cotton [48]. It is well established that the Cry protein concentration in *Bt*-transgenic crops varies with plant variety, plant age, and environmental conditions including temperature, relative humidity, light, and soil properties [51]–[57]. It might thus be useful to study the expression levels and patterns for other *Bt* soybean varieties in other geographical regions where the plants will be grown. The Cry protein concentrations reported also vary with the detection method, including the extraction procedure and the ELISA kit [58]–[60]. Because we used the same methods to analyse all of our samples, the values reported within our study are comparable.

For the assessment of the exposure of non-target species to Cry1Ac toxin in *Bt* soybean fields, it is important to determine which arthropod species are likely to be exposed to the toxin under field conditions and at what level. We thus collected a total of 52 species or taxa belonging to 42 families in 10 different arthropod orders from *Bt* soybean fields and measured their Cry1Ac content.

Among herbivores, no toxin was detected in the soybean aphid *Aphis glycines* (Hemiptera: Aphididae), a species that feeds exclusively on phloem sap. This result agrees with previous reports that phloem feeders ingest little or no Cry protein when feeding on *Bt* plant tissues [23], [61], [62]. In another sap-sucking herbivore, the leafhopper *C. viridis*, significant amounts of Cry1Ac were found in the nymphal stages but not in the adults. Previous studies in *Bt* maize and *Bt* cotton fields also reported a low level of Cry proteins in different species of Cicadellidae [43], [44], [63]. Mesophyll-feeding bugs (Hemiptera) belonging to the Miridae (i.e., *Lygus* spp.) and Pentatomidae (*D. baccarum and H. halys*) contained measurable concentrations of Cry1Ac (between 1 and 10% of the concentration measured in soybean leaves). In contrast, levels detected in *Rhopalus maculates* (Hemiptera: Rhopalidae) were less than 15% of that in leaves. In the case of *Lygus* spp., nymphs contained much higher (5- to 23-fold) concentrations than adults. A similar difference between life stages has been reported for *Lygus rugulipennis* (Hemiptera: Miridae) collected in Cry3Bb1-expressing *Bt* maize [44]. An artificial diet study with *Lygus hesperus* (Hemiptera: Miridae) revealed that only a small portion of the ingested Cry1Ac toxin was absorbed into the hemolymph while most was excreted in a still biologically active form [64]. Field investigations revealed that the abundances of *Lygus* spp., *C. viridis*, and *D. Baccarum* were similar in *Bt* soybean and control soybean (Yu et al., unpublished data). Although these hemipteran pests ingested the Cry1Ac toxin, they did not appear to be adversely affected [65].

In our study, a number of leaf-feeding herbivores contained considerable amounts of Cry1Ac. By far the highest concentration was detected in adults of the grasshopper *A. sinensis* (levels reached 50% of that in soybean leaves), while their nymphs contained amounts of Cry1Ac that were 75- to 8100-times lower. The large difference between the life stages could be explained by the fact that adults ingest significantly more food than nymphs [66], [67]and that adults are apparently inefficient at digesting or excreting the ingested Cry1Ac protein. In contrast, adults of the leaf beetle *P. suturalis nigrobilineatus* (Coleoptera: Chrysomelidae) contained low Cry1Ac toxin concentrations (less than 1% of that in soybean leaves), and the concentrations did not differ among soybean growth stages. This despite the fact that adults of this species are reported to feed on soybean plants [68].The low Cry1Ac concentration detected is surprising given that, in previous studies, adults of *Oulema* species in *Bt* maize fields contained among the highest concentrations found in the sampled arthropods [43], [44]. In the case of Lepidoptera that were analysed in the current study, Cry1Ac was detected at low levels (up to 2% of the concentration in soybean leaves) in larvae, while no toxin was found in the adult stages, regardless of species.

Besides herbivores, a number of common predatory species were collected and analysed in the current study. We found detectable amounts of Cry1Ac in adults and larvae of *Chrysoperla* spp., the larvae of *P. japonica*, the adults and nymphs of *Geocoris pallidipennis* (Hemiptera: Lygaeidae), the adults and nymphs of *Orius* spp., and the nymphs of *Nabis stenoferus* (Hemiptera: Nabidae). The level was highest in *G. pallidipennis* at 0.3 µg/g DW, which was 100-fold lower than the concentration in *Bt* soybean leaves. Overall, our data are similar to those reported for generalist predators in *Bt* cotton, *Bt* rice, and *Bt* maize [41]–[45], [69]. The level of exposure of predatory species to plant-expressed *Bt* Cry proteins is highly variable and can differ depending on the prey consumed, on the time of the last consumption, and on whether the predators have directly consumed plant tissue such as pollen [15]. The predators *Geocoris* spp. and *Nabis* spp., for example, also directly feed on green leaf tissue [70], [71]. Throughout the soybean season, higher levels of Cry1Ac toxin were detected in larvae than in adults of *Chrysoperla* spp. and *P. japonica*. A likely explanation for the difference observed in the case of *Chrysoperla* spp. is the difference in the food consumed. While *Chrysoperla* spp. larvae are predaceous, adults consume pollen, nectar, and aphid honeydew [72]. For *P. japonica*, the situation is less clear because adults and larvae have a similar feeding habit, i.e., both feed on aphids and other small arthropods [73] and also utilize plant pollen and nectar as supplemental food sources [74]. It is thus unclear what caused the difference in Cry1Ac concentration in the two life stages of *P. japonica*. Interestingly, similar results were reported from a field study with Cry1Ab-expressing *Bt* maize in that field-collected larvae of the spider mite predator *Stethorus punctillum* (Coleoptera: Coccinellidae) contained about three-times more Cry protein than adults [43]. Even under controlled laboratory conditions where *S. punctillum*was fed *ad libitum* with *Bt* maize-reared *T. urticae*, the larvae contained significantly more Cry protein than the adults [20]. Anthocorids such as *Orius* spp. are known to feed on pollen in addition to prey. This is likely why *Orius* spp. collected in flowering *Bt* maize fields contained more Cry protein than specimens collected before or after anthesis [43]. In our study, however, *Orius* spp. and an unidentified Anthocorid species contained higher amounts of Cry1Ac when collected after soybean flowering than during flowering. This suggests that pollen feeding is not very important for these two species in soybean fields. The adults of *Deraeocoris punctulatus* (Hemiptera: Miridae) contained relatively high amounts of Cry1Ac toxin when collected before anthesis, but not during or after anthesis. This might be explained by the fact that *D. punctulatus* adults ingests Cry1Ac when they supplement their diet by feeding on soybean tissues [75]. Adults of the aphid predator *Aphidoletes abietis* (Diptera: Cecidomyiidae) are known to feed on nectar and honeydew [76] and are thus not exposed to the Cry protein.

Predatory spiders such as *M. tricuspidata* and *E. graminicolum* are true generalists and play an important role in controlling pests such as thrips, spider mites, leafhoppers, aphids, and lepidopteran larvae in *Bt* crop fields [77]. In addition to encountering *Bt* toxin when feeding on above-ground herbivores, spiders may also be exposed to *Bt* toxin when feeding on pollen and when feeding on soil-associated prey; the latter may have acquired the toxin as a consequence of root feeding or as a consequence of the exudation of toxin by roots and its subsequent passage through the detrital food web [78], [79]. Despite their contribution to biological control and despite the multitude of pathways through which spiders may be exposed to *Bt* toxins in agroecosystems, few studies have measured the uptake of *Bt* toxin by spiders and their exposure level in the field. In three previous studies with *Bt* maize or *Bt* cotton, field-collected spiders of different families (Linyphiidae, Araneidae, Tetragnathidae, and Theridiidae) were found to contain detectable concentrations of Cry toxin, and the amount of uptake of Cry toxin was associated with their prey spectrum [41], [44], [63]. Generally, the uptake of *Bt* toxin by spiders (Theridiidae and Lycosidae) is low because the toxin is diluted as it is transferred from the first trophic level (*Bt* crops) to the second (prey) and because rapid excretion and digestion likely prevent the toxin from accumulating in spider bodies [33], [40]. In our study, three species of spiders (*M. tricuspidata*, *E. graminicolum*, and an unidentified species of Thomisidae) collected from *Bt* soybean were found to contain Cry1Ac toxin, indicating that exposure pathways exist for these spiders in soybean fields. Although relatively high amounts of Cry1Ac (about 13% of the level in soybean leaves) were found in the unidentified species of Thomisidae, the data should be considered with caution because the analysis was not replicated. Lower levels of Cry1Ac (about 1% of that detected in soybean leaves) were found in adult *M. tricuspidatus* throughout the soybean season, which suggests that *M. tricuspidatus* in *Bt* soybean field is likely to consume prey that contain a similar Cry concentration throughout the season and that pollen consumption is not an important exposure pathway.

## Conclusions

The current report provides the first data concerning the exposure of non-target arthropods to Cry proteins in *Bt* soybean fields. The Cry1Ac concentration detected in arthropods varied among arthropod species/taxa, between arthropod life stages, and among the growth stages of the soybean plants. The highest Cry1Ac concentration, which was about 50% of that in soybean leaves, was found in adults of a grasshopper species. Other herbivorous arthropods that were positive for Cry1Ac contained levels between 1 and 10% of that found in the plants; these included a cicadellid sap-feeder, and a number of hemipteran species that are known to feed on mesophyll tissue, and the adults of a curculionid beetle. Among the predators, the highest concentrations were detected in a thomisid spider and an unidentified species of Anthocoridae. For the remaining species/taxa, concentrations were <1% or even below the detection limits of the ELISA. Such information on the exposure of different arthropod groups to the plant-expressed Cry protein within complex food webs is required for non-target risk assessment. More specifically, such information enables researchers to focus on those species that are most likely to be at risk from the insecticidal compound in *Bt* crops [15], [23], [80], [81].

## Supporting Information

Table S1 Cry1Ac concentrations in arthropods collected in *Bt* soybean before, during, and after anthesis in 2010. ELISA results below the limit of detection (LOD) are indicated as ‘<’ with the corresponding LOD value.
(DOCX)
