# Supplementary material for: Acquisition of Cry1Ac Protein by Non-Target Arthropods in Bt Soybean Fields
Source: PLoS One. 2014 Aug 11;9(8):e103973. doi: 10.1371/journal.pone.0103973 (PMC4128818; doi:10.1371/journal.pone.0103973)
Supplement: Table S1 — Cry1Ac concentrations in arthropods collected in Bt soybean before, during, and after anthesis in 2010. ELISA results below the limit of detection (LOD) are indicated as ‘<’ with the corresponding LOD value. (DOCX) [file pone.0103973.s001.docx]

Table S1. Cry1Ac concentrations in arthropods collected in *Bt* soybean before, during, and after anthesis in 2010. ELISA results below the limit of detection (LOD) are indicated as ‘<’with the corresponding LOD value.

| Order | Family | Species | Stage | Functional group^a^ | Mean dry weight per individual [mg] | Mean Cry1Ac concentration [µg/g] (number of replicates, number of individuals per replicate)^b^ | | |
| --- | --- | --- | --- | --- | --- | --- | --- | --- |
|  |  |  |  |  |  | Before anthesis | During anthesis | After anthesis |
| **Araneae** | Linyphiidae | *Erigonidium graminicolum* (Sundevall) | Adult | P | 0.61 | < 0.009 (1,3) | 0.040(1,7) | < 0.008 (1,2) |
|  | Lycosidae | *Pardosa T-insignita* Bosenberg & Strand | Mix | P | 3.31 | n.c. | <0.010 (2,6) | n.c. |
| **Coleoptera** | Chrysomelidae | *Callosobruchus chinensis* (Linnaeus) | Adult | H | 1.18 | n.c. | <0.002 (1,1) | 0.023 (1,1) |
|  | Curculionidae | *Xylinophorus mongolicus* Zumpt, T. | Adult | H | 1.30 | n.c. | n.c. | 0.269 (1,1) |
|  |  | *Sympiezomias velatus* Kôno, H. | Adult | H | 2.45 | n.c. | 0.318 (1,2) | n.c. |
|  | Elateridae | *Pleonomus canaliculatus* Falderman | Adult | H | 2.51 | n.c. | <0.011 (1,2) | n.c. |
|  | Tenebrionidae | Not identified | Adult | H | 2.40 | n.c. | n.c. | <0.011 (1,1) |
| **Dermaptera** | Anisolabididae | *Euborellia pallipes* Shiraki | Adult | O | 2.33 | n.c. | <0.003 (1,1) | n.c. |
| **Diptera** | Agromyzidae | *Melanagromyza sojae* Zehntner | Adult | H | 0.53 | 0.026 (1,7) | n.c. | n.c. |
|  | Anthomyiidae | *Delia platura* (Meigen) | Adult | H | 0.46 | n.c. | <0.007 (1,5) | n.c. |
|  | Calliphoridae | Not identified | Adult | H | 4.10 | n.c. | n.c. | <0.003 (1,5) |
|  | Dolichopodidae | Not identified | Adult | H | 1.94 | n.c. | <0.004 (1,3) | <0.001 (2,2-5) |
|  | Drosophilidae | Not identified | Adult | H | 0.53 | 0.04 (1,5) | <0.003 (2,11) | 0.105 (2,8-10) |
|  | Syrphidae | *Episyrphus balteatus* (De Geer) | Adult | H | 6.30 | n.c. | <0.001 (1,2) | <0.003 (1,3) |
|  |  | *Sphaerophoria* sp. | Adult | H | 2.60 | n.c. | n.c. | <0.001 (1,1) |
| **Hemiptera** | Alydidae | *Riptortus pedestris* (Fabricius) | Adult | O | 52.3 | n.c. | n.c. | 1.496 (1,1) |
|  |  |  | Nymph | O | 20.1 | n.c. | <0.004 (1,1) | 3.236 (1,2) |
|  | Pentatomidae | *Eysacoris guttiger* (Thunberg) | Adult | H | 13.9 | n.c. | n.c. | <0.003 (1,1) |
| **Hymenoptera** | Apidae | *Apis mellifera ligustica* Spinola | Adult | H | 17.3 | 0.040 (1,1) | <0.002 (1,1) | n.c. |
|  | Braconidae | *Microplitis mediator* (Haliday) | Adult | H | 1.15 | n.c. | <0.009 (2,5) | <0.005 (1,4) |
|  | Formicidae | Not identified | Adult | P | 0.95 | n.c. | <0.008 (2,5) | <0.004 (1,6) |
|  | Ichneumonidae | *Campoletis chlorideae* Uchida | Adult | H | 10.6 | n.c. | n.c. | <0.006 (1,1) |
|  | Sphecidae | Not identified | Adult | H | 11.4 | n.c. | <0.002 (2,1) | n.c. |
|  | Vespidae | Not identified | Adult | P | 7.20 | n.c. | n.c. | <0.009 (1,1) |
| **Lepidoptera** | Arctiidae | *Spilosoma niveus* (Ménétriés) | Adult | H | 62.1 | <0.002 (2,1-2) | <0.0004 (2,2) | n.c. |
|  |  |  | Larva | H | 25.8 | 0.570 (2,2) | n.c. | n.c. |
|  | Lycaenidae | *Plebejus argus* (Linnaeus) | Adult | H | 1.80 | n.c. | n.c. | <0.008 (1,1) |
|  | Pieridae | *Colias poliographus* Motschulsky | Adult | H | 3.70 | n.c. | n.c. | <0.002 (1,1) |
|  | Pyralidae | *Dichocrosis punctiferalis* Guenée | Adult | H | 4.81 | <0.015 (1,2) | n.c. | n.c. |
|  | Sphingidae | *Clanis bilineata tsingtauica* Mell | Larva | H | 70.0 | n.c. | n.c. | 0.027 (2,1) |
| **Odonata** | Zygoptera | Not identified | Adult | P | 8.15 | n.c. | n.c. | 1.060 (1,2) |
| **Orthoptera** | Acrididae | *Diabolocatantops pinguis* (Stål) | Adult | H | 84.5 | 0.070 (2,1) | n.c. | n.c. |
|  |  |  | Nymph | H | 35.4 | 0.040 (2,1) | n.c. | n.c. |

^a^ H – herbivore, P – predator, O – omnivore

^b^ n.c. – not collected
